# HMGB1 Exacerbates Intestinal Barrier Damage by Inducing Ferroptosis Through the TLR4/NF-κB/GPX4 Pathway in Ulcerative Colitis

**DOI:** 10.1155/mi/2395557

**Published:** 2025-07-11

**Authors:** Nannan Zhu, Xiaoyuan Ge, Lixue Zhang, Xinwen Chen, Weizhen Xiang, Qiao Mei

**Affiliations:** Department of Gastroenterology, The First Affiliated Hospital of Anhui Medical University, Hefei City, Anhui Province, China

**Keywords:** ferroptosis, HMGB1, intestinal barrier, TLR4/NF-κB/GPX4 pathway, ulcerative colitis

## Abstract

**Background:** Intestinal barrier dysfunction and persistent inflammatory response are key pathophysiologic features of ulcerative colitis (UC). High mobility group box-1 protein (HMGB1), an important inflammatory mediator and immunomodulatory factor, has been shown to be involved in the pathogenesis of UC. However, the association between HMGB1 and intestinal barrier dysfunction is unclear.

**Methods:** In this study, we investigated the mechanism of HMGB1 driving intestinal barrier damage by integrating clinical data, animal models, and cellular experiments in UC. First, HMGB1 levels and its correlation with intestinal barrier protein expression in UC patients were verified based on Gene Expression Omnibus (GEO) dataset GSE75214 analysis and western blotting (WB) assay. Subsequently, colitis was induced in C57BL/6J mice using dextran sodium sulfate (DSS) and intervened with dipotassium glycyrrhizinate (DPG), and the effects of HMGB1 on colonic inflammation, ferroptosis, and intestinal barrier were assessed by histopathological scoring, qRT-PCR, enzyme-linked immunosorbent assay (ELISA), WB assay, immunofluorescence, and transmission electron microscopy (TEM) examination. Lastly, the influence of HMGB1 on ferroptosis-related genes expression, TLR4/NF-κB/GPX4 pathway activation and intestinal barrier damage were revealed by transepithelial electrical resistance (TEER) value measures, FITC-dextran permeability detections, qRT-PCR, and WB assays in vitro Caco-2 cell models.

**Results:** HMGB1 expression was significantly elevated in colonic tissues of UC patients (especially in active stage), and was negatively correlated with barrier protein expression. In DSS-induced colitis mouse model, HMGB1 upregulation accompanied by changes in TLR4, NF-κB, and GPX4 expression and ferroptosis-related genes upregulation, while inhibition of HMGB1 attenuated inflammation, restored barrier function, and reversed ferroptosis. Moreover, cellular experiments further confirmed HMGB1 induced ferroptosis and intestinal barrier damage in Caco-2 cells via the TLR4/NF-κB/GPX4 pathway.

**Conclusion:** Our results suggest that HMGB1 drives ferroptosis through the TLR4/NF-κB/GPX4 signaling pathway, thereby exacerbating intestinal inflammation and barrier damage in UC. Targeting this pathway may provide a novel therapeutic strategy for UC.

## 1. Introduction

Ulcerative colitis (UC) is a chronic nonspecific inflammatory bowel disease (IBD) characterized by diffuse, recurrent inflammation of the intestinal tract [1]. Recently, the incidence of IBD has increased rapidly in many newly industrialized countries, including China [2]. Approximately, 20%–30% of UC patients will eventually require surgery due to complications and ineffective conservative treatment as the disease progresses [3]. Although the etiology of UC has not been fully elucidated, numerous studies have shown that impairment of the intestinal mucosal barrier is a key part of the pathogenesis of UC [4, 5]. Intestinal mucosal barrier is centered on polarized intestinal epithelial cells (IECs) that maintain monolayer continuity through apical polarity complexes (PAR3/PAR6/aPKC), and its paracellular permeability is synergistically regulated by tight junction (TJ) and adherens junctions (AJ) [6, 7]. TJ rely on Claudin and Occludin to achieve a charge selective barrier, whereas AJ mediate homophilic adhesion during epithelial injury via E-cadherin and β-catenin [8]. Emerging evidence suggests that persistent intestinal barrier dysfunction contributes to abnormal colonization of intestinal microbiota and hyperactivation of immune system, driving the transition from acute inflammation to chronic tissue damage in UC [9].

High mobility group box-1 protein (HMGB1) is a nonhistone molecule with both intranuclear homeostatic regulation and extracellular inflammatory signaling functions [10]. HMGB1 participates in DNA repair by stabilizing nucleosome structure in physiological state, yet is actively secreted or passively released extracellularly in response to cellular stress or necrosis, and acts as a damage-associated molecular pattern (DAMP) to trigger innate immune responses. Clinical cohort studies have confirmed that serum and intestinal mucosal levels of HMGB1 are significantly elevated in patients with UC, with correlations to disease severity and poor prognosis [11, 12]. Researches demonstrated that HMGB1 activates NF-κB by binding to Toll-like receptor 4 (TLR4) and induces a massive release of inflammatory mediators, thus, exacerbating intestinal epithelial barrier disruption and immune cell infiltration [13]. In dextran sodium sulfate (DSS)-induced acute colitis model, histopathologic damage and inflammatory factor storm in mouse colon were markedly attenuated after blocking the interaction of HMGB1 with TLR4 using a specific inhibitor (dipotassium glycyrrhizinate, DPG) [14, 15]. All of the above reveal that HMGB1 is a key mediator in driving intestinal inflammatory cascade response.

Recently, ferroptosis has been confirmed as a critical factor leading to epithelial damage in UC [16], which is characterized by glutathione peroxidase 4 (GPX4) inactivation, lipid peroxidation, and mitochondrial dysfunction [17]. In contrast to apoptosis or necrosis, the typical morphological changes of ferroptosis include mitochondrial atrophy, cristae structural disintegration, and reactive oxygen species (ROS) accumulation [18]. Molecular regulatory network of ferroptosis involves the synergistic action of multiple genes such as key genes for energy metabolism (ATP synthase F0 complex subunit C3, ATP5G3); lipid peroxidation markers (prostaglandin endoperoxide synthase 2, PTGS2); rate-limiting enzyme of the tricarboxylic acid cycle (citrate synthase, CS); iron homeostasis regulators (iron-responsive element-binding protein 2, IREB2); and ribosomal protein L8 (RPL8), and other key genes [19]. Remarkably, ferroptosis-induced oxidative stress contributes to loosening of intestinal epithelial intercellular junctions and increased barrier permeability by affecting TJ protein expression [20]. Recent studies point to activation of inflammation-related signaling pathways causing ferroptosis [21]. Zhou et al. [22] revealed the intense association of HMGB1 with redox imbalance and mitochondrial dysfunction, processes that are closely related to nonapoptotic cell death modes such as ferroptosis. On this basis, we hypothesized that HMGB1 might be involved in regulating the process of ferroptosis and intestinal barrier damage.

Therefore, our study established DSS-induced colitis models and Caco-2 monolayer cell models to test our hypothesis, reveal the molecular mechanism of HMGB1-induced ferroptosis and intestinal barrier damage, provide potential therapeutic targets for UC treatment.

## 2. Material and Methods

### 2.1. Chemicals and Reagents

DSS salt (9011-18-1) was purchased from Santa Ana MP Biomedical, California, USA. DPG (HY-N0184A), HMGB1 (HY-P70570), TAK-242 (HY-11109), and Ferrostatin-1 (Fer-1, HY-100579) were purchased from MedChemExpress, USA. FITC-Dextran (GC19938 and GC36048) was purchased from GLPBIO, USA. Anti-β-ACTIN (66009-1-Ig), anti-HMGB1 (10829-1-AP), anti-Occludin (27260-1-AP), anti-Claudin-1(28674-1-AP), anti-TLR4 (66350-1-Ig), and anti-GPX4 (67763-1-Ig) antibodies were purchased from Proteintech Biotechnology, China. Anti-E-Cadherin (14472T) and Na,K-ATPase α1 (52387) antibodies were purchased from Danvers Cell signal technology, USA. Anti-NF-κB p65 (T55034) and anti-Phospho-NF-κB p65 (TP56372) antibodies were purchased from Abmart Pharmaceuticals Technology Ltd., China.

### 2.2. Bioinformatics Analysis of UC Patients

Microarray data derived from the Gene Expression Omnibus (GEO) database (GSE75214), comprising 97 patients with UC, 22 controls, and 75 patients with CD, were used for bioinformatics analysis. Raw data were systematically processed and deep analyzed and images created using R software. To present the transcriptome level differences of HMGB1 gene in control and UC samples, GraphPad Prism software was applied to produce violin plots. The “pROC” and “ggplot2” packages were employed to construct ROC curves and correlation scatter plots. Differentially expressed genes (DEGs) were mapped as heatmaps using “pheatmap” package, and volcano plots associated with DEGs were constructed by “ggplot2” package. Functional enrichment analysis is visually presented via histograms and bubble plots created by the R package “clusterProfiler.”

### 2.3. Collection of Colon Tissue Samples

In this study, colonic samples were gathered from 10 patients with active UC, and 10 healthy subjects from the Department of Gastroenterology of the First Affiliated Hospital of Anhui Medical University. Obtained colon samples are placed into precooled cryopreservation tubes immediately, flash frozen in liquid nitrogen, then stored at -80°C freezer for subsequent analyses. The study strictly adhered to ethical norms with approval from the Ethics Committee of the Hospital (PJ2022-07-27). Each participant's written consent was obtained before the beginning of the study.

### 2.4. DSS-Induced Colitis

Male C57BL/6J mice (age: 6–8 weeks old, body weight: 18–22 g) were purchased from Jiangsu Jicui PharmaTech Co., Ltd. (Production License No.: SCXK (Su) 2023-0009). During the experimental period, the mice adapted to eat and drink freely, and raised for 1 week at 22°C under a 12 h light/12 h dark cycle. Twenty-four mice were randomly divided into four groups: control group (*n* = 6), DSS group (*n* = 6), DPG group (*n* = 6), and DSS + DPG group (*n* = 6). The experiment lasted for 7 days, during which sufficient food supply was guaranteed. The control group was given free water; the DSS group was given 3% DSS in drinking water to induce acute colitis; the DPG group was given 8 mg/kg/day of DPG in drinking water; and the DSS + DPG group was given both 3% DSS and 8 mg/kg/day of DPG in drinking water. Body weight, fecal traits, and occult blood were monitored and recorded daily in four groups of mice to evaluate the disease activity index (DAI). All experimental procedures strictly followed the guidelines of the Laboratory Animal Center of Anhui Medical University.

### 2.5. Histological Analysis

Experimental animals were executed by cervical dislocation on day 8 of the trial. Subsequently, colon tissue was excised immediately and measured for colon length. To examine the histopathologic alterations in mouse colon tissues, 4% paraformaldehyde solution was used to fix the colon tissues. Then paraffin embedding, sectioning (4 μm thickness), and hematoxylin–eosin (HE) staining were processed serially. Sections were observed under a light microscope for histologic scoring according to previously described criteria [23].

### 2.6. Immunofluorescence Staining Analysis

Sections of mouse colon tissue were warmed at 60°C for 1 h to melt the paraffin wax, then dewaxed and hydrated in xylene and ethanol. Subsequently, citrate solution was used to repair the antigen and immunostaining sealer for 1 h. Sections were incubated with primary antibodies (HMGB1, 1:200; TLR4 1:200; Na,K-ATPase α1,1:200; Occludin, 1:200; E-cadherin, 1:500; and Claudin-1,1:200) at 4°C overnight. Then incubate with the corresponding secondary antibodies (1:200) for 1 h, respectively. After DAPI staining, observing the slides using confocal microscopy. ImageJ software was applied to quantitatively analyze the obtained images.

### 2.7. Transmission Electron Microscopy (TEM) Examination

Colon tissues collected from DSS-induced colitis mice were sliced into 1 mm^3^ narrow strips, and then sequentially fixed in 2.5% glutaraldehyde and 1% osmium tetroxide solution to maintain ultrastructural integrity. Then, gradient ethanol and acetone dehydration and epoxy resin immersion embedding were performed sequentially. Thin sections were cut using an ultrathin sectioning machine and stained with uranyl acetate and lead citrate. Finally, intestinal epithelial TJs and mitochondrial morphology were observed using a transmission electron microscope (JEM1400, Japan).

### 2.8. Cell Culture and Treatment

This study was conducted in human colorectal adenocarcinoma cell line Caco-2 acquired from the cell bank of the Chinese Academy of Sciences. Caco-2 cells were incubated in DMEM medium with 10% fetal bovine serum at 37°C with 5% CO_2_. Replaced the cell culture solution every 1–2 days. Cells passaged to 3–6 generations are prepared for cellular experimentation. To confirm the role of the HMGB1-TLR4 pathway, cells were grouped into (1) control group: medium culture for 24 h; (2) HMGB1 group: stimulation with HMGB1 (100 ng/mL) for 24 h; (3) HMGB1+TAK-242 group: 2 h pretreatment with the TLR4-specific inhibitor TAK-242 (10 μM) followed by combined HMGB1 (100 ng/mL) stimulation for 24 h; (4) HMGB1 +Fer-1 group: pretreatment with the ferroptosis inhibitor Fer-1(2 μM) for 2 h, followed by HMGB1 (100 ng/mL) combined HMGB1 (100 ng/mL) stimulation for 24 h. All cell experiments were repeated three times to ensure reliability of the results.

### 2.9. Transepithelial Electrical Resistance (TEER) Measurement

Caco-2 cells were plated on polycarbonate membrane inserts (12 mm diameter, 3 μm pore size; Corning, USA) at a density of 4.0×10^5^ cells per transwell and cultured until monolayers formed, with medium changes every 48 h. Monolayer cells with a steady-state resistance value of at least 500 Ω were selected for the experiments. Cellular interventions take the previously described approach. Resistance measurements are performed using a strictly calibrated and sterilized EVOM2 (World Precision Instruments, USA). The resistance was tested at three random points in the upper chamber and lower chamber for each transwell, averaged, and then calculated according to the following formula: TEER (Ω. cm^2^) = (Ω sample − Ω blank) ×*S* (membrane area). Blank control was a cell-free transwell system. To accurately reflect changes in cellular barrier function, TEER results were normalized to initial baseline values before treatment.

### 2.10. Paracellular Permeability Assay

Caco-2 cells were inoculated into transwell inserts until monolayers formed, and then treated with different chemical agents for 24 h. Replace the upper chamber medium with 4 and 10 kDa FITC-dextran (1 mg/mL), respectively, and incubate with monolayers cells for 1 h. Then, fluorescence intensity of the lower chamber medium was detected using a microplate reader at an excitation at 495 nm and an emission at 525 nm. According to relative fluorescence units (RFU), FITC-dextran concentrations were calculated against a standard curve, and the results were normalized to the initial baseline value before treatment.

### 2.11. Quantitative Real-Time PCR (qRT-PCR)

Total RNA was abstracted from Caco-2 cells and mouse colon samples, then reverse transcribed into cDNA. The primers were synthesized by Shanghai Sangon Biotechnology Co., Ltd. with reference to the primer sequences of mouse and human genes in the gene bank (detailed primer sequences are shown in Table 1). qRT-PCR was conducted using ChamQ SYBR qPCR Master Mix. Applying 2^−*ΔΔ*CT^ method to compare the expression levels of different genes with β-actin as the internal reference gene.

### 2.12. Western Blot (WB)

Proteins extracted from human colon samples, mouse colon samples, and Caco-2 cells. Specific procedures as follows: put the sample in the configured protein lysate, lysis on ice for 30 min, high-speed centrifugation at 4°C, 12,000 rpm for 15 min, take up the supernatant and mix with appropriate loading buffer, boil, and denaturation. SDS-PAGE was utilized to isolate equivalent proteins, then transferred to the PVDF membrane. The PVDF membranes were blocked in rapid blocking buffer for 1 h, and then incubated overnight at 4°C with specific primary antibodies namely, anti-β-actin (1:5000), anti-HMGB1 (1:5000), anti-Occludin (1:2000), anti-E-Cadherin (1:2000), anti-TLR4 (1:1000), anti-GPX4 (1:1000), anti-Claudin-1(1:2000), anti-NF-κB p65 (1:1000), and anti-Phospho-NF-κB p65 (1:1000). Next, incubate the membrane with enzyme-labeled secondary antibody (1:10,000) for 1 h at room temperature. Imaging of proteins on PVDF membranes by chemiluminescence detection and quantitative analysis of digital images by ImageJ software.

### 2.13. Enzyme-Linked Immunosorbent Assay (ELISA)

The level of inflammatory cytokines (TNF-α, IL-1β, IL-6, and IL-17) in mouse colon tissue samples and cell culture supernatants was determined using an ELISA kits (Jiangsu Enzyme Immunity Industry Co., Ltd., China) according to the instructions provided by the manufacturer.

### 2.14. Statistical Analysis

Statistical analysis and data visualization utilizing SPSS 22.0 and GraphPad Prism 8.0 software. The data satisfying the normal distribution are expressed as $\overset{―}{x}\pm s$, otherwise as median (IQR). Comparison between two groups were made by *t*-test, while one-way ANOVA was used for comparisons between three groups, and post hoc comparisons by LSD test. Considered statistically significant at *p* < 0.05.

## 3. Results

### 3.1. HMGB1 Expression Was Upregulated in UC

**Figure 1:**
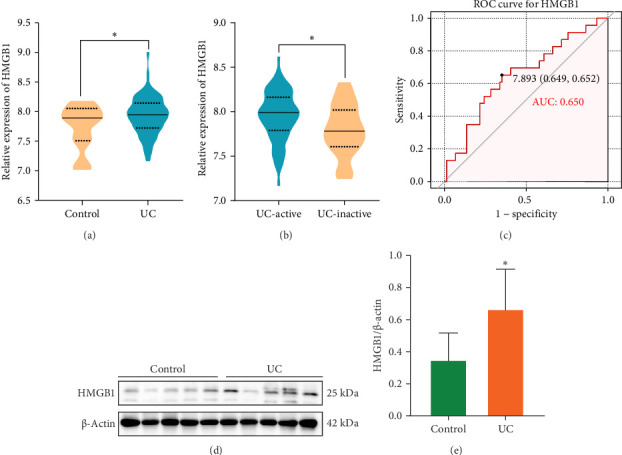
HMGB1 expression was increased in the intestinal tissues of patients with UC. (A) Relative gene expression of HMGB1 in colonic tissues of UC patients compared with controls (using dataset GSE75214; controls, *n* = 22; UC, *n* = 97). (B) Relative gene expression of HMGB1 in colonic tissues of active compared with remission UC patients (using dataset GSE75214; remission, *n* = 23; active, *n* = 74). (C) ROC curve analysis of HMGB1 for assessing disease activity in GSE75214. (D) HMGB1 expression in colonic tissues of UC patients was detected by Western blot (*n* = 10). (E) Quantitative analysis results of (D). Data are presented as mean±SD (error bars). *⁣*^*∗*^*p* < 0.05.

Based on bioinformatic methods, this study analyzed the GSE75214 dataset included in GEO to identify HMGB1 expression in colonic tissues of UC patients. Results suggested that HMGB1 was significantly upregulated in UC patients compared to controls, especially in patients with active UC (Figure 1A,B). The area under the ROC curve for HMGB1 was 0.65, demonstrating its potential to predict UC disease activity (Figure 1C). To validate these findings, WB detection of clinical biopsies confirmed significant elevation of HMGB1 protein levels in patients with UC (Figure 1D,E).

### 3.2. HMGB1 Aggravates the Inflammatory Response

**Figure 2:**
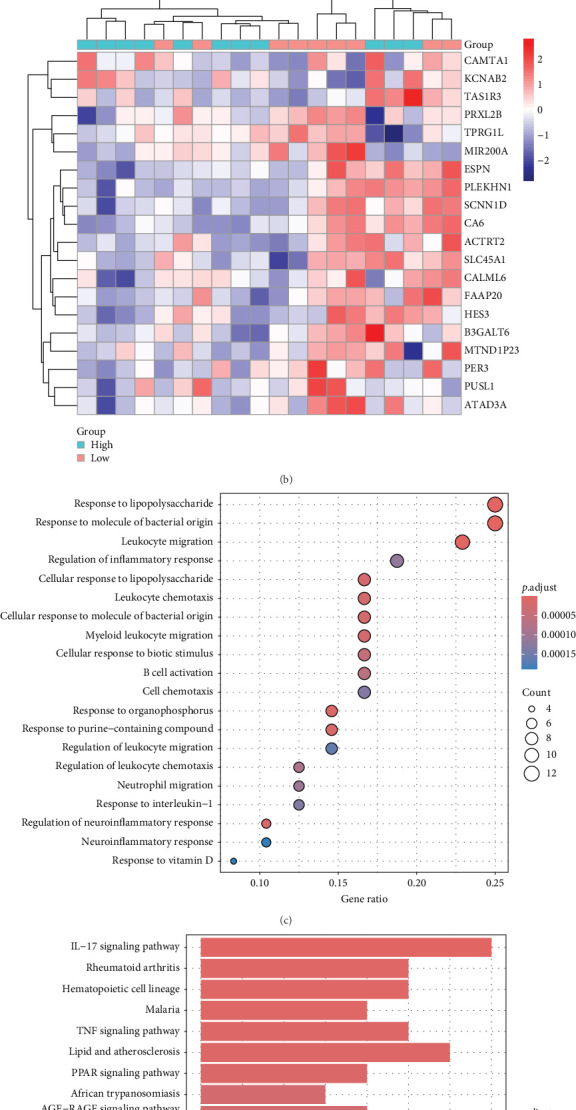
HMGB1 regulates the inflammatory response. (A) Volcano map of HMGB1 related DEGs in UC patients (GSE75214). (B) Heatmap of the top 20 genes of HMGB1 related DEGs (GSE75214). (C) GO functional enrichment analysis of DEGs. (D) KEGG pathway enrichment analysis of DEGs.

To clarify the effect of HMGB1 in UC pathogenesis, we divided colon tissue samples from UC patients in the GSE75214 dataset into low and high expression groups based on the median value of HMGB1. According to differential expression screening criteria (fold change≥1.0, *p*-value<0.05), 51 significant DEGs were identified by DESeq2 algorithm analysis, of which 18 genes were upregulated and 33 genes were downregulated in expression. The volcano map presents a complete distribution pattern of DEGs (Figure 2A), and the heatmap shows the top 20 genes with the most significant differences (Figure 2B). Remarkably, GO functional enrichment indicated these DEGs were mainly involved in the regulation of inflammatory responses (e.g., “leukocyte migration” and “lipopolysaccharide response”) (Figure 2C), while KEGG pathway analyses revealed that they were closely associated with the activation of Toll-like receptor, NF-κB, and IL-17 signaling pathways (Figure 2D).

**Figure 3:**
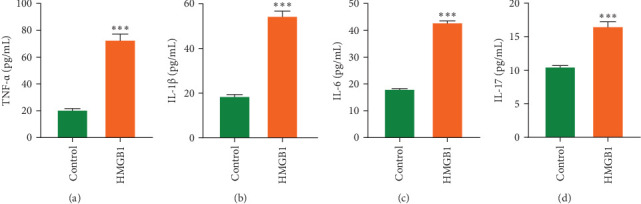
HMGB1 promotes inflammatory factor release in Caco-2 cells. Caco-2 cells were stimulated with HMGB1 (100 ng/mL) for 24 h followed by ELISA to detect the concentrations of: (A) TNF-α, (B) IL-1β, (C) IL-6, and (D) IL-17 (*n* = 3). Data are presented as mean±SD (error bars). *⁣*^*∗*^*p* < 0.05, *⁣*^*∗∗*^*p* < 0.01, and *⁣*^*∗∗∗*^*p* < 0.001 vs. Control group.

**Figure 4:**
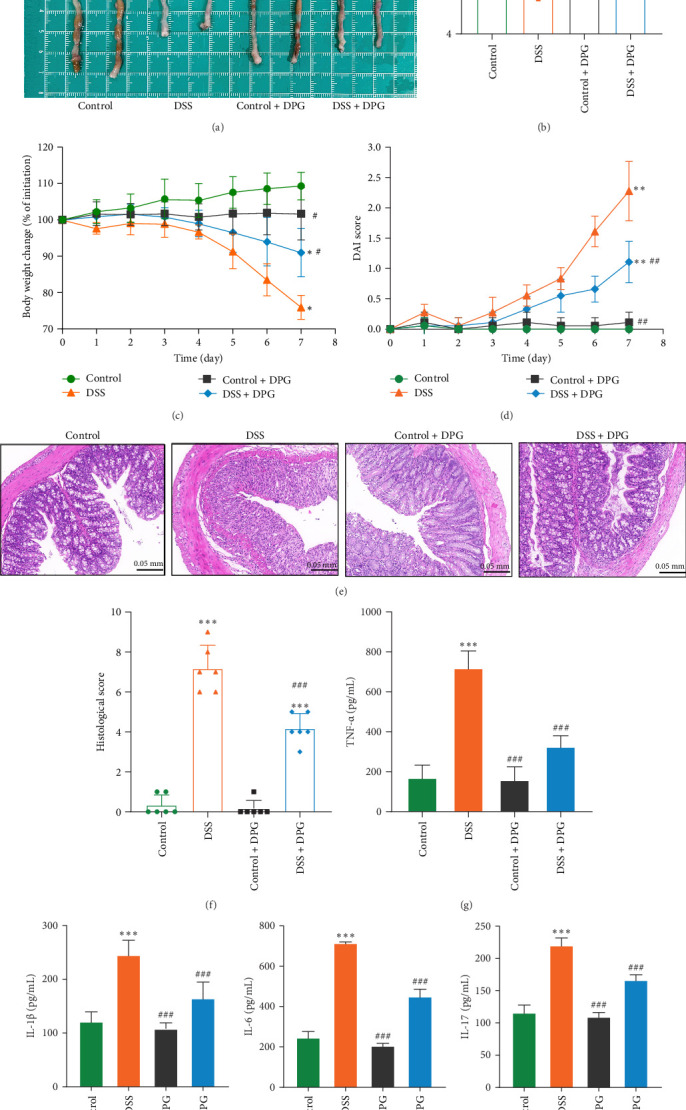
HMGB1 aggravates DSS-induced colitis in mice. (A) Changes in colonic length of mice in each group (*n* = 6). (B) Quantitative analysis results of (A). (C) Changes in body weight of mice in each group (*n* = 6). (D) Changes in DAI scores of mice in each group (*n* = 6). (E) HE staining to observe the damage of colonic tissue in mice (*n* = 6). (F) Quantitative analysis results of (E). (G–J) Concentrations of TNF-α, IL-1β, IL-6, and IL-17 detected using ELISA (*n* = 6). Data are presented as mean ± SD (error bars). *⁣*^*∗*^*p* < 0.05, *⁣*^*∗∗*^*p* < 0.01, and *⁣*^*∗∗∗*^*p* < 0.001 vs. Control group; ^#^*p* < 0.05, ^##^*p* < 0.01, and ^###^*p* < 0.001 vs. DSS group.

Taking into account, the HMGB1 mediated inflammatory regulatory network revealed by bioinformatics analysis, this study carried out functional validation by constructing a Caco-2 cell inflammation model and a DSS-induced colitis model in mice. In the Caco-2 cell model, the concentrations of inflammatory mediators TNF-α, IL-1β, IL-6, and IL-17 in the cell culture supernatants were markedly elevated after 24 h of stimulation with HMGB1 (100 ng/mL) (Figure 3A–D). However, the concentrations of above inflammatory mediators were significantly decreased in colonic tissues of DSS-induced colitis mice compared to the DSS group after intervention with DPG, a HMGB1 specific inhibitor (Figure 4G–J). Importantly, inhibition of HMGB1 ameliorated body weight loss, shortened colon length, DAI scores, and histopathological damage in DSS-induced colitis mice (Figure 4A–F).

### 3.3. HMGB1 is Associated With Intestinal Barrier Damage

**Figure 5:**
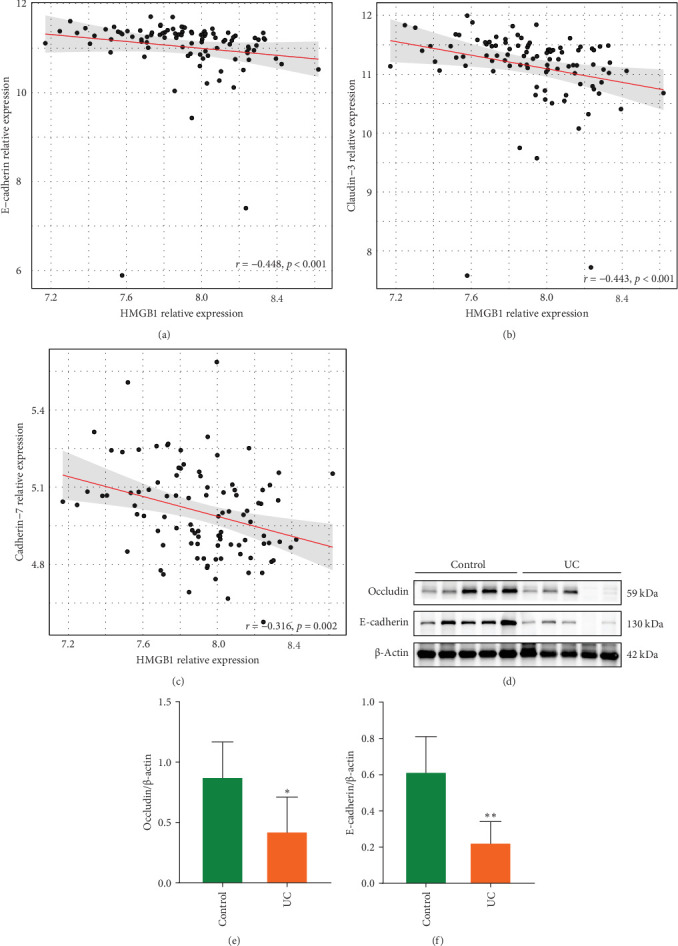
HMGB1 associated with intestinal barrier protein expression. (A) Spearman rank correlation analysis between HMGB1 and mRNA levels of E-cadherin (A), Claudin-3 (B), and Cadherin-7(C). (D) Expression levels of Occludin and E-cadherin proteins in colonic tissues of control and UC patients were evaluated by western blot (*n* = 10). (E) Quantitative analysis of Occludin protein. (F) Quantitative analysis of E-cadherin protein. Data are presented as mean ± SD (error bars). *⁣*^*∗*^*p* < 0.05 and *⁣*^*∗∗*^*p* < 0.01 vs. Control group.

**Figure 6:**
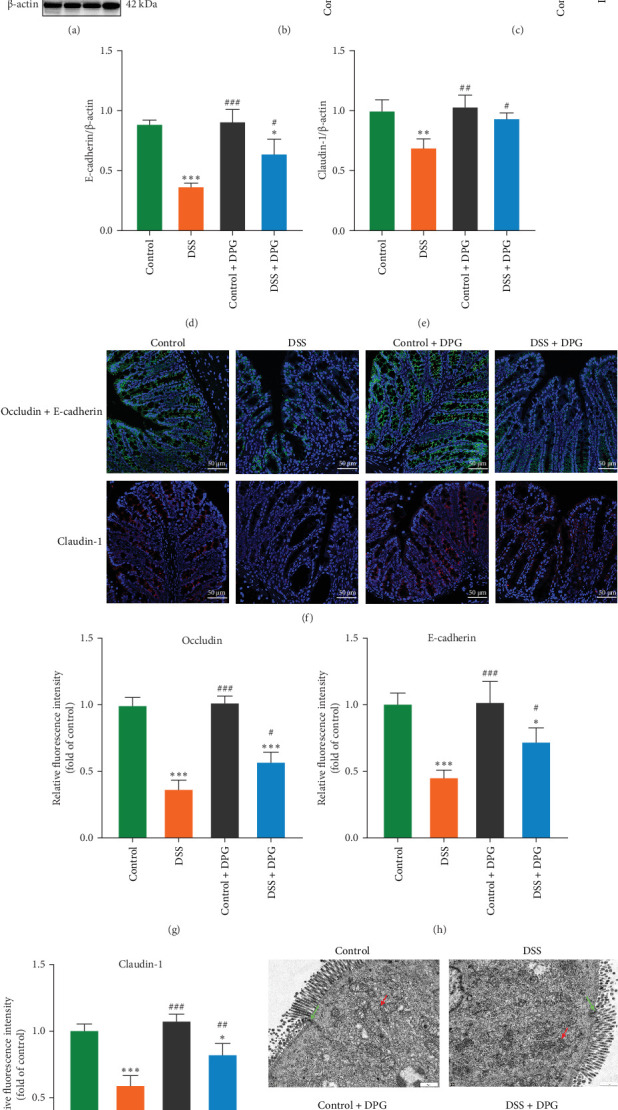
HMGB1 damages intestinal barrier in colitis mice. (A) The effect of HMGB1 on expression of Occludin, E-cadherin, and Claudin-1 proteins in mouse colon tissues was assessed by western blot (*n* = 6). (B) Quantitative analysis of HMGB1 (B), Occludin (C), E-cadherin (D), and Claudin-1(E) proteins expression in different groups (*n* = 6). (F) Representative immunofluorescence images of Occludin, E-cadherin, and Claudin-1 in the mice colonic tissues of each group (*n* = 6). (G) Quantitative analysis on fluorescence intensity of Occludin (G), E-cadherin (H), and Claudin-1 (I) proteins expression in different groups (*n* = 6). (J) Ultrastructural alterations of the intestinal barrier and mitochondria observed by TEM. Green arrows indicate tight junctions and red arrows indicate mitochondria. Data are presented as mean ± SD (error bars). *⁣*^*∗*^*p* < 0.05, *⁣*^*∗∗*^*p* < 0.01, and *⁣*^*∗∗∗*^*p* < 0.001 vs. Control group; ^#^*p* < 0.05, ^##^*p* < 0.01 and ^###^*p* < 0.001 vs. DSS group. Note: Immunofluorescence staining results are shown as synthesized images. DAPI: blue, Occludin: red, E-cadherin: green, Claudin-1: red.

**Figure 7:**
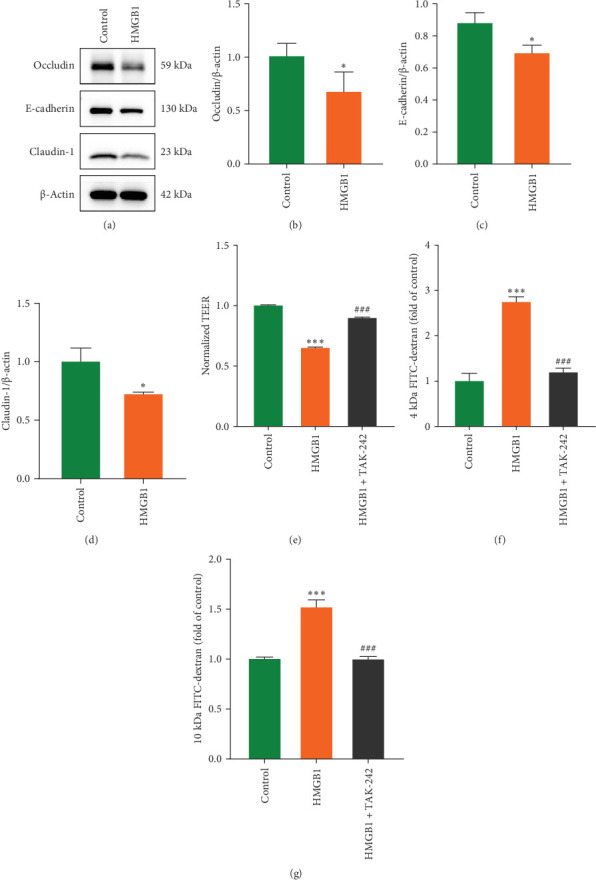
HMGB1 destroys the barrier function of Caco-2 cells in vitro. (A) The effect of HMGB1 on expression of Occludin, E-cadherin, and Claudin-1 proteins in Caco-2 cells was assessed by western blot (*n* = 3). (B) Quantitative analysis of Occludin (B), E-cadherin (C), Claudin-1(D) proteins expression in different groups (*n* = 3). (E) TEER values (E), 4 kDa (F) and 10 kDa (G) FITC-dextran flux were determined in the presence or absence of HMGB1 and TAK-242 stimulation for 24 h (*n* = 3). Data are presented as mean ± SD (error bars). *⁣*^*∗*^*p* < 0.05, and *⁣*^*∗∗∗*^*p* < 0.001 vs. Control group; ^###^*p* < 0.001 vs. HMGB1 group.

We further looked into the effect of HMGB1 on UC intestinal barrier damage. Analysis based on the GSE75214 dataset revealed that HMGB1 negatively correlated with the expression of important barrier proteins (E-cadherin, Claudin-3, Cadherin-7) in colon tissues of UC patients (Spearman's correlation coefficients of -0.448, -0.443, and -0.316, respectively) (Figure 5A–C), and WB results further confirmed the downregulation of Occludin and E-cadherin protein levels in the colonic tissues of UC patients (Figure 5D–F). In the DSS-induced colitis model, HMGB1 overexpression was accompanied by downregulation of the barrier proteins Occludin, E-cadherin, and Claudin-1, which was reversed by DPG intervention, as further corroborated by immunofluorescence staining results (Figure 6A–I). TEM ultrastructural analyses showed obvious shortening of intestinal villi and widening of the TJs gaps between IECs in the DSS group, whereas suppressing HMGB1 greatly improved structural disorders of intestinal villi and restored the TJs (Figure 6J). Moreover, in vitro experiments likewise proved the detrimental impact of HMGB1 on expression of Occludin, E-cadherin, and Claudin-1 in Caco-2 cells (Figure 7A–D), as well as resulting in lower TEER values and higher FITC permeability (Figure 7E–G). Altogether, these results demonstrate the damaging role of HMGB1 on the integrity of intestinal epithelial barrier.

### 3.4. HMGB1 Induces Ferroptosis in Intestinal Epithelial Cells

**Figure 8:**
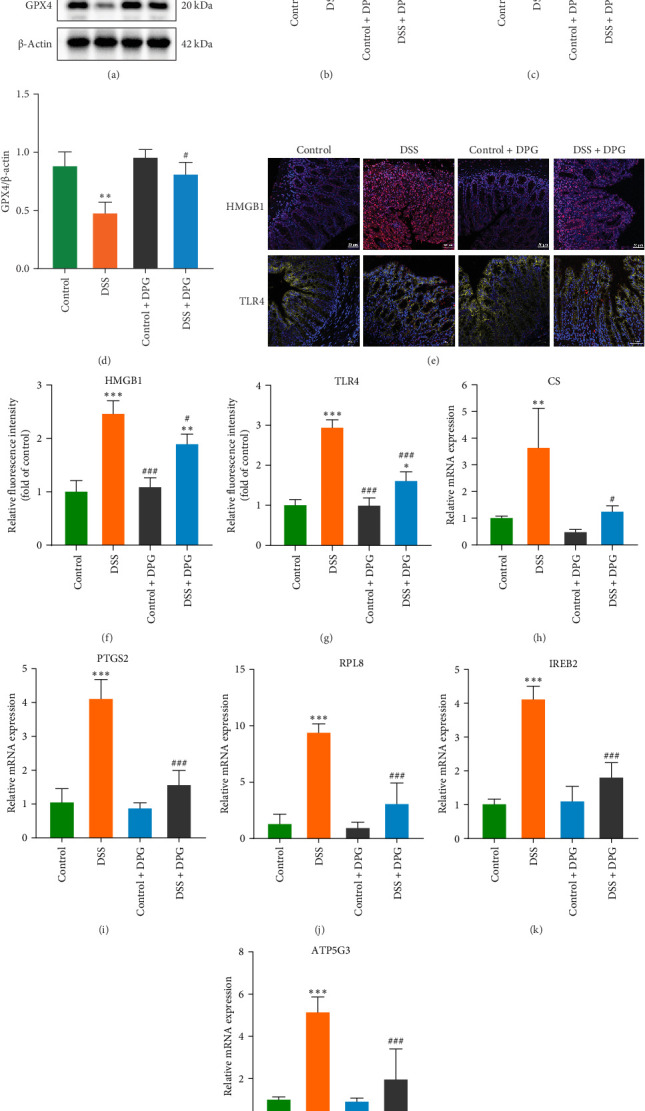
HMGB1 regulates ferroptosis through TLR4/NF-κB/GPX4 signaling pathway in colitis mice. (A) Variations of TLR4, NF-κB, p-NF-κB, and GPX4 proteins expression in mouse colon tissues from various groups detected by western blot (*n* = 6). (B) Quantitative analysis of TLR4 (B), p-NF-κB (C), and GPX4 (D) proteins expressions in different groups (*n* = 6). (E) Representative immunofluorescence images of HMGB1 and TLR4 in the mice colonic tissues of each group (*n* = 6). (F) Quantitative analysis on fluorescence intensity of HMGB1 (F) and TLR4 (G) proteins expression in different groups (*n* = 6). (H) The mRNA levels of (H) CS, (I) PTGS2, (J) RPL8, (K) IREB2, and (L) ATP5G3 were detected by qRT-PCR in mouse colon tissues from different groups (*n* = 6). Data are presented as mean±SD (error bars). *⁣*^*∗*^*p* < 0.05, *⁣*^*∗∗*^*p* < 0.01, and *⁣*^*∗∗∗*^*p* < 0.001 vs. Control group; ^#^*p* < 0.05, ^##^*p* < 0.01, and ^###^*p* < 0.001 vs. DSS group. Note: Immunofluorescence staining results are shown as synthesized images. DAPI: blue, HMGB1: red, Na, K-ATPase: yellow, TLR4: red.

Ferroptosis, a form of programed cell death characterized by GPX4 inactivation and mitochondrial dysfunction, has been shown to be intimately associated with intestinal barrier damage [20]. To verify whether HMGB1 mediated intestinal barrier damage is associated with ferroptosis, we conducted a systematic study in a DSS-induced colitis model. As a results of WB assay, GPX4 expression was significantly decreased in colonic tissues of mice in the DSS group, whereas GPX4 expression was elevated in the DSS + DPG group (Figure 8A). Notably, qRT-PCR results revealed the mRNA levels of ferroptosis-related genes (CS, PTGS2, RPL8, IREB2, and ATP5G3) were significantly upregulated in the DSS group and decreased in the DSS + DPG group (Figure 8H–L). In addition, colonic tissue mitochondria of mice in the DSS group presented typical pathological alterations as observed by TEM, manifested by reduced mitochondrial cristae density, volume reduction, and vacuolization, whereas DPG treatment significantly restored the mitochondrial ultrastructure of colitis mice (Figure 6J), indicating an essential role of HMGB1 in inducing ferroptosis in IECs.

### 3.5. HMGB1 Induces Ferroptosis Through the TLR4/NF-*κ*B/GPX4 Signaling Pathway

**Figure 9:**
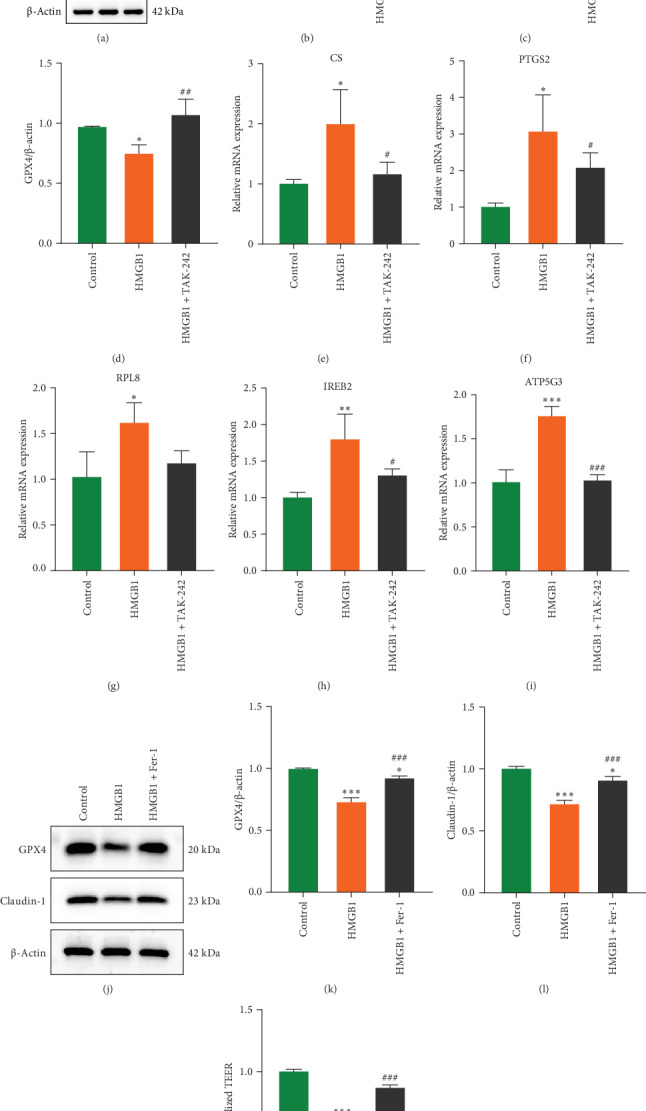
HMGB1 regulates ferroptosis via the TLR4/NF-κB/GPX4 signaling pathway of Caco-2 cells in vitro, mediating barrier damage. (A) Variations in TLR4, NF-κB, p-NF-κB, and GPX4 proteins expression in Caco-2 cells were detected by Western blot in the presence or absence of HMGB1 and TAK-242 stimulation for 24 h (*n* = 3). (B) Quantitative analysis of TLR4 (B), p-NF-κB (C), and GPX4 (D) proteins expressions in different groups (*n* = 3). (E) The mRNA levels of (E) CS, (F) PTGS2, (G) RPL8, (H) IREB2, and (I) ATP5G3 were detected by qRT-PCR in Caco-2 cells from different groups (*n* = 3). (J) Variations in GPX4 and Claudin-1 proteins expression in Caco-2 cells were detected by western blot in the presence or absence of HMGB1 and Fer-1 stimulation for 24 h (*n* = 3). (K) Quantitative analysis of GPX4 (K) and Claudin-1 (L) proteins expressions in different groups (*n* = 3). (M) TEER values were determined in the presence or absence of HMGB1 and Fer-1 stimulation for 24 h (*n* = 3). Data are presented as mean±SD (error bars). *⁣*^*∗*^*p* < 0.05, *⁣*^*∗∗*^*p* < 0.01, and *⁣*^*∗∗∗*^*p* < 0.001 vs. Control group; *⁣*^#^*p* < 0.05, *⁣*^##^*p* < 0.01, and *⁣*^###^*p* < 0.001 vs. HMGB1 group.

Previous studies have demonstrated that HMGB1 modulates inflammatory responses through activation the TLR4/NF-κB signaling pathway, while GPX4, a key regulator of ferroptosis, possibly regulated by TLR4 signaling in its expression [24, 25]. Consequently, we speculate HMGB1 may exacerbate intestinal barrier damage by inducing ferroptosis in IECs through TLR4/NF-κB/GPX4 signaling pathway. Our results showed that the expression levels of TLR4 and p-NF-κB in the colon tissues of mice in the DSS group were significantly increased, while the expression of GPX4 presented a decreasing trend. After DPG treatment, the levels of TLR4 and p-NF-κB were effectively suppressed, and the expression of GPX4 was restored, as shown in Figure 8A–D. Immunofluorescence staining also confirmed this result (Figure 8E–G). In addition, HMGB1 treatment significantly promoted the expression of TLR4 and p-NF-κB proteins in Caco-2 cells, downregulated GPX4 protein levels (Figure 9A–D), and was accompanied by notable upregulation of mRNAs of ferroptosis-related genes (CS, PTGS2, RPL8, IREB2, and ATP5G3) (Figure 9E–I). Treatment with TAK-242 reverses the above phenomenon. Notably, GPX4, barrier protein Claudin-1 and TEER values were restored in Caco-2 cells when ferroptosis inhibitor Fer-1 added (Figure 9J–M). These results suggest that HMGB1 mediates intestinal barrier damage by regulating ferroptosis through the TLR4/NF-kB/GPX4 pathway.

## 4. Discussion

UC is a chronic recurrent IBD involving dysregulation of mucosal immune homeostasis and disruption of intestinal epithelial barrier integrity; however, the core molecules driving this pathological cascade response have not been fully defined. In this study, we observed HMGB1 expression significant upregulation in patients with UC and DSS-induced colitis models in mice. By facilitating release of inflammatory mediators, disrupting barrier proteins (e.g., E-cadherin, Occludin, and Claudin-1) and triggering intestinal epithelial cell ferroptosis, HMGB1 damages the intestinal epithelial barrier. Mechanistically, HMGB1 mediates IECs ferroptosis and barrier disruption through activation of the TLR4/NF-kB/GPX4 signaling pathway.

HMGB1 is a highly conserved nuclear protein that serves as DAMP to drive immune activation and chronic inflammation when released extracellularly in response to stressors [13]. Current studies have clearly revealed TLR4 to be the core regulatory hub of HMGB1 extracellular function, after binding to TLR4, HMGB1 promotes inflammatory factor storm via MyD88-dependent NF-κB pathway [26]. Palone et al. [27] demonstrated that fecal HMGB1 was strongly correlated with endoscopic activity (assessed by MES) in both pediatric and adult patients with UC. Our study discovered HMGB1 expression markedly upregulated in colonic tissues of UC patients, and strongly correlated with endoscopic disease activity. Consistent with previous findings [28], higher levels of HMGB1 were observed in DSS-induced colitis models in mice concomitant with weight loss, shortened colon length, elevated DAI scores, and aggravation of pathologic damage. In addition, cellular experiments further confirmed the proinflammatory effects of HMGB1, as evidenced by a prominent increase in inflammatory factors TNF-α, IL-1β, IL-6, and IL-17. These results support that HMGB1 involved in disease progression of UC.

Defective and impaired intestinal mucosal barrier function has been shown to be a characteristic change in UC [29, 30]. Integrity of intestinal mucosal barrier relies on synergistic regulation of TJ and AJ. The TJ central protein Occludin forms a charge selective pore with the Claudin family, and maintains barrier function through ZO-1 anchoring to the cytoskeleton; and the AJ critical protein E-cadherin regulates epithelial polarity via Ca^2+^ dependent dimerization and the β-catenin signaling axis. Remarkably, in the inflammatory microenvironment, epigenetic reprograming and posttranscriptional regulation together drive dysregulation of barrier protein expression. IL-1β targets degradation of Occludin mRNA through upregulation of MIR200C-3p leading to increased TJ permeability [31], whereas IL-6 induces epithelial mesenchymal transition (EMT) and barrier disintegration by stimulating ribosomal biogenesis to downregulate p53 expression and decrease E-cadherin expression [32]. Kojima et al. [33] suggested that highly expressed HMGB1 relates to enhanced epithelial permeability, and our investigation further demonstrates the critical effect of HMGB1 in the regulation of intestinal barrier function. The GSE75214 microarray data revealed negative correlation between HMGB1 levels and barrier protein expression, which is consistent with the results of WB assay for HMGB1 in colon samples from patients with UC. In a DSS-induced colitis model, inhibition of HMGB1 was observed to increase intestinal Occludin, E-cadherin, and Claudin-1 expression in experimental mice. Besides, the results of WB assay, TEER, and paracellular permeability assay confirmed the deleterious impact of HMGB1 stimulation on barrier protein expression in Caco-2 cells. These findings suggest that HMGB1 promotes the disintegration of intercellular TJ, thereby disrupting intestinal barrier function.

To clarify the specific molecular mechanism involved in the impairment of intestinal barrier function by HMGB1, this study identified that HMGB1 associated DEGs were significantly enriched in TLR4 and NF-*κ*B signaling pathway through analyzing the GSE75214 dataset. Our study verified the inducing effect of HMGB1 on TLR4, p-NF-KB expression in both in vivo and in vitro models. Current studies have revealed that p-NF-KB could induce ferroptosis by decreasing the transcriptional process of antioxidant molecules (e.g., GPX4, NQO1, and HMOX1), which in turn induces ferroptosis [21]. Different from cell necrosis and pyroptosis, ferroptosis is characterized by iron accumulation, lipid peroxidation, ROS generation, and GPX4 depletion as the main biochemical features [34]. In particular, GPX4 is the main antioxidant target of ferroptosis and specifically eliminates lipid peroxides from cell membranes by catalyzing glutathione-dependent reduction reactions, thereby maintaining membrane structural integrity [34]. In a DSS-induced colitis model, we observed that activation of the HMGB1/TLR4/NF-kB pathway resulted in decreased GPX4 expression, accompanied by increased mRNA levels of ferroptosis-related indicators (PTGS2, IREB2, CS, RPL8, and ATP5G3), which was further verified by cellular experiments. PTGS2 increased expression and release has been considered to be highly correlated with ferroptosis, and studies concluded that PTGS2 exacerbates cellular damage by promoting arachidonic acid metabolism and release of inflammatory factors during ferroptosis [35]. Furthermore, elevated levels of IREB2 are closely related to the accumulation of unstable iron pools and lipid peroxidation [36]. We also found that the induction of ferroptosis and barrier damage in Caco-2 cells by HMGB1 was significantly inhibited using Fer-1. Our results suggest that HMGB1 affects ferroptosis in IECs through the TLR4/NF-kB/GPX4 signaling pathway to mediate intestinal barrier damage.

Remarkably, HMGB1 and ferroptosis are affected by multiple factors. For instance, contrast media can significantly increase intracellular and serum HMGB1 levels [37]. Benzyl alcohol attenuated acetaminophen-induced liver injury in mice, the underlying mechanism of which may be closely related to the inhibition of the TLR4 signaling pathway and consequently the reduction of HMGB1 release [38]. Furthermore, diets rich in polyunsaturated fatty acids exacerbate the course of IBD by inducing ferroptosis through accelerated lipid peroxidation processes [39]. Increased production of free radicals as well as decreased functioning of antioxidant defense systems in long-term chronic inflammatory states further contribute to the onset and progression of ferroptosis [40]. Future studies should tightly control these variables and explore in depth their potential interactions with HMGB1 and ferroptosis in UC.

Despite this study revealing a crucial role for HMGB1 in UC, several limitations still remain. First, the sample size of colonic tissues from UC patients is relatively limited, and although we have further validated this with a mouse model of colitis, validation in a wider population is needed to ensure the generalizability of results. Second, although the present study elucidated a novel mechanism by which HMGB1 induces ferroptosis through the TLR4/NF-kB/GPX4 pathway leading to intestinal barrier damage, HMGB1 may also affect the intestinal barrier function by mediating other types of cell death, and the specific mechanism of its action needs to be further explored. Last, inhibition of HMGB1 ameliorated intestinal inflammation in a mouse model of colitis, however, how to target HMGB1 safely and effectively in the clinic still requires further investigation.

In conclusion, our study demonstrated that HMGB1 mediates ferroptosis and intestinal barrier damage through the TLR4/NF-kB/GPX4 signaling pathway in UC. Inhibition of HMGB1 has potential to be an effective therapeutic strategy and provide new insights into the clinical management of UC.

## Figures and Tables

**Table 1:** Primer sets used for qRT-PCR.

Gene	Forward primer (5′-3′)	Reverse primer (5′-3′)
mACTIN	GGCTGTATTCCCCTCCATCG	CCAGTTGGTAACAATGCCATGT
mCS	GGACAATTTTCCAACCAATCTGC	AGTCAATGGCTC CGATACTGC
mPTGS2	TTCCAATCCATGCAAAACCGT	AGTCCGGGTACAGTCACACTT
mRPL8	ACGTGAAGCACCGTAAGGG	GATGCCTTTAATGTAGCCGTGT
mIREB2	CGGCACCAAGTATGATATTCTGC	AGGGCACTTCAACATTGCTCT
mATP5G3	GTTTCAGACCAGTGTAATCAGCA	AGAACCAGCAACTCTACTGT
hACTIN	CCTGGCACCCAGCACAAT	GGGCCGGACTCGTCATCA
hCS	TGCTTCCTCCACGAATTTGAAA	CCACCA TACATCATGTCCACAG
hPTGS2	CTGGCGCTCAGCCATACAG	CGCACTTATACTGGTCAAATCCC
hRPL8	AAGGGCATCGTCAAGGACATC	CAGCTCCGTCCGCTTCTTAAA
hIREB2	TCGATGTATCTAAACTTGGCACC	GCCATCACAATTTCGTACAGCAG
hATP5G3	CCAGAGTTGCATACAGACCAAT	CCCATTAAATACCGTAGAGCCT

## Data Availability

The data that support the findings of this study are available upon reasonable request.

## References

[1] Ordás I., Eckmann L., Talamini M., Baumgart D. C., Sandborn W. J. (2012). Ulcerative Colitis. *The Lancet*.

[2] Cui G., Yuan A. (2018). A Systematic Review of Epidemiology and Risk Factors Associated With Chinese Inflammatory Bowel Disease. *Frontiers in Medicine*.

[3] Costa J., Magro F., Caldeira D., Alarcão J., Sousa R., Vaz-Carneiro A. (2013). Infliximab Reduces Hospitalizations and Surgery Interventions in Patients With Inflammatory Bowel Disease. *Inflammatory Bowel Diseases*.

[4] van der Post S., Jabbar K. S., Birchenough G. (2019). Structural Weakening of the Colonic Mucus Barrier Is an Early Event in Ulcerative Colitis Pathogenesis. *Gut*.

[5] Porter R. J., Kalla R., Ho G. T. (2020). Ulcerative Colitis: Recent Advances in the Understanding of Disease Pathogenesis. *F1000Research*.

[6] Buckley C. E., St Johnston D. (2022). Apical-Basal Polarity and the Control of Epithelial Form and Function. *Nature Reviews Molecular Cell Biology*.

[7] Soranno D. E., Coopersmith C. M., Brinkworth J. F. (2025). A Review of Gut Failure as a Cause and Consequence of Critical Illness. *Critical Care*.

[8] Naser A. N., Lu Q., Chen Y. H. (2023). Trans-Compartmental Regulation of Tight Junction Barrier Function. *Tissue Barriers*.

[9] Zhang Z., Li D., Zheng S., Zheng C., Xu H., Wang X. (2025). Gene Expression Regulation and Polyadenylation in Ulcerative Colitis via Long-Chain RNA Sequencing. *BMC Genomics*.

[10] Bianchi M. E., Crippa M. P., Manfredi A. A., Mezzapelle R., Rovere Querini P., Venereau E. (2017). High-Mobility Group Box 1 Protein Orchestrates Responses to Tissue Damage via Inflammation, Innate and Adaptive Immunity, and Tissue Repair. *Immunological Reviews*.

[11] Foelsch K., Pelczar P., Zierz E. (2024). Intestinal Epithelia and Myeloid Immune Cells Shape Colitis Severity and Colorectal Carcinogenesis Via High-Mobility Group Box Protein 1. *Journal of Crohn’s and Colitis*.

[12] Chen Y. M., Wu D., Sun L. J. (2020). Clinical Significance of High-Mobility Group Box 1 Protein (HMGB1) and Nod-Like Receptor Protein 3 (NLRP3) in Patients With Ulcerative Colitis. *Medical Science Monitor*.

[13] Liu H., Liao X., Zhang Z. (2025). HMGB1: Key Mediator in Digestive System Diseases. *Inflammation Research*.

[14] Khan U., Karmakar B. C., Basak P. (2023). Glycyrrhizin, an Inhibitor of HMGB1 Induces Autolysosomal Degradation Function and Inhibits Helicobacter Pylori Infection. *Molecular Medicine*.

[15] Lu B., Wang C., Wang M. (2014). Molecular Mechanism and Therapeutic Modulation of High Mobility Group Box 1 Release and Action: An Updated Review. *Expert Review of Clinical Immunology*.

[16] Niu R., Lan J., Liang D. (2024). GZMA Suppressed GPX4-Mediated Ferroptosis to Improve Intestinal Mucosal Barrier Function in Inflammatory Bowel Disease. *Cell Communication and Signaling*.

[17] Stockwell B. R. (2022). Ferroptosis Turns 10: Emerging Mechanisms, Physiological Functions, and Therapeutic Applications. *Cell*.

[18] Newton K., Strasser A., Kayagaki N., Dixit V. M. (2024). Cell Death. *Cell*.

[19] Zhu K., Zhu X., Liu S., Yu J., Wu S., Hei M. (2022). Glycyrrhizin Attenuates Hypoxic-Ischemic Brain Damage by Inhibiting Ferroptosis and Neuroinflammation in Neonatal Rats via the HMGB1/GPX4 Pathway. *Oxidative Medicine and Cellular Longevity*.

[20] Zhu J., Wu Y., Zhang L. (2024). Epithelial Piezo1 Deletion Ameliorates Intestinal Barrier Damage by Regulating Ferroptosis in Ulcerative Colitis. *Free Radical Biology and Medicine*.

[21] Chen Y., Fang Z.-M., Yi X., Wei X., Jiang D.-S. (2023). The Interaction Between Ferroptosis and Inflammatory Signaling Pathways. *Cell Death & Disease*.

[22] Zhou X. R., Wang X. Y., Sun Y. M. (2024). Glycyrrhizin Protects Submandibular Gland Against Radiation Damage by Enhancing Antioxidant Defense and Preserving Mitochondrial Homeostasis. *Antioxidants & Redox Signaling*.

[23] Kihara N., de la Fuente S. G., Fujino K., Takahashi T., Pappas T. N., Mantyh C. R. (2003). Vanilloid Receptor-1 Containing Primary Sensory Neurones Mediate Dextran Sulphate Sodium Induced Colitis in Rats. *Gut*.

[24] Zhu K., Fan R., Cao Y. (2024). Glycyrrhizin Attenuates Myocardial Ischemia Reperfusion Injury by Suppressing Inflammation, Oxidative Stress, and Ferroptosis Via the HMGB1-TLR4-GPX4 Pathway. *Experimental Cell Research*.

[25] Jing X., Zhou G., Zhu A., Jin C., Li M., Ding K. (2024). RG-I Pectin-Like Polysaccharide from *Rosa chinensis* Inhibits Inflammation and Fibrosis Associated to HMGB1/TLR4/NF-*κ*B Signaling Pathway to Improve Non-Alcoholic Steatohepatitis. *Carbohydrate Polymers*.

[26] Sims G. P., Rowe D. C., Rietdijk S. T., Herbst R., Coyle A. J. (2010). HMGB1 and RAGE in Inflammation and Cancer. *Annual Review of Immunology*.

[27] Palone F., Vitali R., Cucchiara S. (2014). Role of HMGB1 as a Suitable Biomarker of Subclinical Intestinal Inflammation and Mucosal Healing in Patients With Inflammatory Bowel Disease. *Inflammatory Bowel Diseases*.

[28] Chen X., Bao S., Liu M. (2024). Inhibition of HMGB1 Improves Experimental Mice Colitis by Mediating NETs and Macrophage Polarization. *Cytokine*.

[29] Sánchez de Medina F. D., Romero-Calvo I., Mascaraque C., Martínez-Augustin O. (2014). Intestinal Inflammation and Mucosal Barrier Function. *Inflammatory Bowel Diseases*.

[30] Neurath M. F., Travis S. P. L. (2012). Mucosal Healing in Inflammatory Bowel Diseases: A Systematic Review. *Gut*.

[31] Rawat M., Nighot M., Al-Sadi R. (2020). IL1B Increases Intestinal Tight Junction Permeability by Up-Regulation of MIR200C-3p, Which Degrades Occludin mRNA. *Gastroenterology*.

[32] Brighenti E., Calabrese C., Liguori G. (2014). Interleukin 6 Downregulates p53 Expression and Activity by Stimulating Ribosome Biogenesis: A New Pathway Connecting Inflammation to Cancer. *Oncogene*.

[33] Kojima T., Shindo Y., Konno T. (2022). Dysfunction of Epithelial Permeability Barrier Induced by HMGB1 in 2.5D Cultures of Human Epithelial Cells. *Tissue Barriers*.

[34] Jin X., Tang J., Qiu X. (2024). Ferroptosis: Emerging Mechanisms, Biological Function, and Therapeutic Potential in Cancer and Inflammation. *Cell Death Discovery*.

[35] Yang W. S., SriRamaratnam R., Welsch M. E. (2014). Regulation of Ferroptotic Cancer Cell Death by GPX4. *Cell*.

[36] Xia H., Wu Y., Zhao J. (2023). N6-Methyladenosine-Modified circSAV1 Triggers Ferroptosis in COPD Through Recruiting YTHDF1 to Facilitate the Translation of IREB2. *Cell Death & Differentiation*.

[37] Yuan J., Guo L., Ma J. (2024). HMGB1 as an Extracellular pro-Inflammatory Cytokine: Implications for Drug-Induced Organic Damage. *Cell Biology and Toxicology*.

[38] Cai C., Huang H., Whelan S. (2014). Benzyl Alcohol Attenuates Acetaminophen-Induced Acute Liver Injury in a Toll-Like Receptor-4-Dependent Pattern in Mice. *Hepatology*.

[39] Mayr L., Grabherr F., Schwärzler J. (2020). Dietary Lipids Fuel GPX4-Restricted Enteritis Resembling Crohn’s Disease. *Nature Communications*.

[40] Ye Y., Liu L., Jing Y. (2024). Ferroptosis: A Therapeutic Opportunity of Inflammatory Bowel Disease. *Chinese Medical Journal*.

